# A longitudinal and case-control study of dropout among drug users in methadone maintenance treatment in Haiphong, Vietnam

**DOI:** 10.1186/s12954-017-0185-7

**Published:** 2017-08-30

**Authors:** Pham Minh Khue, Nguyen Thi Tham, Dinh Thi Thanh Mai, Pham Van Thuc, Vu Minh Thuc, Pham Van Han, Christina Lindan

**Affiliations:** 1 Haiphong University of Medicine and Pharmacy, 72A, Nguyen Binh Khiem, Ngo Quyen, Haiphong, Vietnam; 2 The State Council for Professor Title of Vietnam, 1, Dai Co Viet, Bach Khoa, Hai Ba Trung, Hanoi, Vietnam; 3 Global Health Sciences, University of California, UCSF, 550 16th Street, Third Floor, San Francisco, CA 94158 USA

**Keywords:** Drug use, Methadone maintenance treatment, Opioid substitution, Dropout, Vietnam, HIV

## Abstract

**Background:**

Vietnam began providing methadone maintenance therapy (MMT) in 2008; as of June 2016, 44,479 persons who inject drugs (PWID) were in treatment in 57 provinces. However, 10–23% of patients were estimated to have dropped out of treatment during the first 2 years. We evaluated dropout and factors associated with quitting treatment.

**Methods:**

We followed clients ≥ 18 years old enrolled in five MMT clinics in Haiphong for 3 years. Persons who missed a consecutive month of methadone treatment were considered to have dropped out and were not allowed to return; those who missed greater than five consecutive doses were considered to be non-compliant but were allowed to restart treatment at their initial dose. Clients who dropped out or who were non-compliant during their third year of MMT (cases) were traced and matched with two clients who remained in treatment (controls) by gender, age, and length of time in the program. Cases and controls were interviewed. Additional data on levels of yearly retention were abstracted from clinic records.

**Results:**

Among the 1055 patients initially enrolled in MMT, dropout and non-compliance combined was 13.6% during the first year, 16.5% during the second year, and 22.3% during the third year. By 36 months, 33.3% of clients had dropped out, of whom 10.6% had died and 24% had been arrested. We traced and interviewed 81 clients who dropped out or who were non-compliant during year 3 as well as 161 controls. The primary reasons for dropping out included claiming no dependence on heroin (22.2%), conflict with work (21.0%), health problems (16.0%), and inability to afford the methadone co-payment of approximately 0.5 USD/day (14.8%). Independent factors associated with non-compliance included continuing to use heroin (aOR = 12.4, 95% CI 4.2–36.8) and missing greater than three doses during the previous 3 months (aOR = 18.5, 95% CI 7.4–47.1); receiving a daily dose of > 120 mg of methadone was associated with a lower odds ratio of dropping out (aOR = 0.3, 95% CI 0.1–0.9).

**Conclusion:**

By 3 years, one third of all patients in treatment had permanently dropped out. Ensuring that methadone dosing is adequate and reducing or eliminating the co-payment fee for those who cannot afford it could improve retention.

## Background

People who inject drugs (PWID) account for more than half of all people infected with HIV in Vietnam [1, 2]. The HIV prevalence among those who inject drugs is estimated to be 33% and ranges from 3 to 58% in different provinces [3]. Methadone maintenance therapy (MMT) became available in Vietnam in 2008, with the first pilot clinics opening in Haiphong and Ho Chi Minh City [4]. MMT has been shown to reduce transmission of HIV and other blood-borne infections, improve the health and quality of life of those addicted to opiates, and support reintegration into the community [5–8].

The total number of PWID in Vietnam is estimated to be around 260,000, about 10,000 of whom reside in Haiphong, the third largest city in the country [9]. Between 2008 and 2010, more than 2500 PWID in Haiphong were enrolled in the three available MMT clinics [10]; in 2011, five new clinics were opened, and since then, 1055 additional clients have started treatment [11]. Pregnant women, persons who are HIV infected, and those who have families that support their enrolment are given preference to enroll in MMT programs [4, 12]. Haiphong is divided into 14 districts, and patients can only receive methadone in the district in which they or their family are registered. Clients are required to attend the clinic daily to receive their dose. After 1 year of treatment, patients can start reducing their dose in an effort to discontinue methadone, but most continue on maintenance therapy [12].

Initial data from the MMT pilot program in Haiphong and Ho Chi Minh City showed a promising impact of methadone on individual patients’ lives [7], and the government hoped that greater availability of treatment could help limit HIV transmission in the country as a whole [13]. In June 2013, 61 methadone clinics were providing treatment to nearly 14,000 drug users; the Ministry of Health set a target of treating 80,000 drug users by 2015 [4]. To help finance this expansion, clients at many clinics were required to pay for a substantial part of MMT services. In Haiphong, for example, clients pay 10,000 VND (about 0.50 USD) daily or nearly 15 USD every month [8, 14].

An evaluation of Haiphong’s MMT program in 2008 revealed that dropout rates were low, only about 6% during the first 6 months of treatment [15] and much less than among MMT clients in the nearby countries of China and Malaysia [16–18]. However, later unpublished data from clinics indicated that dropout rates were higher, from 10 to 23% during the first 2 years, although still less than in other countries. Nevertheless, a more detailed understanding of factors associated with retention would substantially contribute to the development of appropriate interventions to improve the MMT program in Vietnam. Therefore, we performed a longitudinal study of clients in MMT clinics in Haiphong to measure retention rates over a 3-year period and to identify reasons for dropping out.

## Methods

### Study subjects and sampling

We evaluated patients enrolled from August 2011 to July 2012 in five newly opened MMT clinics in Haiphong and followed them for up to 36 months. The Vietnamese national methadone treatment policy states that clients must obtain methadone at the clinic site daily; those who miss more than five consecutive doses but less than 30 in 1 month are allowed to return for treatment but have to restart methadone at the initiation dose. Those who miss more than 30 consecutive days are considered to have dropped out and are not allowed to re-enroll in a drug substitution program.

We performed two different evaluations. Based on clinic records, we first determined the number and proportion of clients each year who discontinued treatment for more than five consecutive doses and of those who permanently dropped out. Secondly, we identified and attempted to trace those clients who had completed 2 years of treatment but during their third year had either permanently dropped out or had missed more than five consecutive doses. We did not trace clients who dropped out during their first 2 years of MMT, because we were interested in factors associated with longer term retention.

After 2 years of MMT, 819 clients remained in the program. During the subsequent 12 months, 68 clients missed more than five consecutive doses but had returned to treatment, and 115 clients had permanently dropped out; we attempted to locate all 183 of these clients. For each client who had either dropped out or had been non-compliant, and who could be traced and interviewed (case), we identified two clients who remained in treatment throughout their third year of MMT, matching by sex, age, and period of initial enrolment (controls).

Individual interviews of clients who dropped out and who were retained were conducted by research staff at a private location separate from the MMT clinic. The questionnaire included structured items about demographics, use of heroin during the last month of MMT, other drug use, distance from home to the clinic, and whether the client had friends who were using opiates, among other questions. Cases were also asked to respond to close-ended questions about reasons why they had dropped out of treatment. Information on most recent methadone dose, results of hepatitis C and B and HIV testing, and anti-retroviral treatment was abstracted from clinic records.

### Analysis

Data were analyzed in Stata (v. 10.0). The proportion of clients who missed more than five consecutive doses and who permanently dropped out during each 12-month period was calculated. Characteristics of those who dropped out during their third year of MMT and of controls were analyzed using frequency distributions. Factors associated with a combined dropout variable (either missing more than five doses or permanently dropping out) between 24 and 36 months of treatment were determined using univariate logistic regression. Factors associated with the combined dropout variable at *p* ≤ 0.20 on univariate analysis were included in a multivariable logistic regression model. Model fitness was assessed by using the linktest and the Hosmer-Lemeshow test.

### Ethical approval

The protocol was approved by the Committee on Human Subjects of the University of California, San Francisco; the Institutional Review Board of the Haiphong University of Medicine and Pharmacy; and the US Centers for Disease Control Global AIDS Program Associate Director of Science. Cases and controls provided written informed consent to be interviewed and to have their medical records abstracted.

## Results

Among 1055 clients initially enrolled, 111 (10.5%) permanently dropped out and 32 (3.0%) missed greater than five doses during the first year of MMT (Table 1). Among the 944 patients who were on treatment at the start of the second year, 124 (13.1%) dropped out and 32 (3.4%) missed more than five doses during the following 12 months; 819 clients were in treatment at 24 months, of whom 115 (14.0%) dropped out and 68 (8.3%) missed more than five consecutive doses during the subsequent year. Over the 3-year period, a total of 350 (33.2%) clients had permanently dropped out. Reasons for dropout that were recorded on clinic records are shown in Table 1. A total of 37 (10.6%) had died, 84 (24.0%) had been arrested, 25 (7.1%) had transferred to another clinic, and reasons were unknown for 138 (39.4%).

**Table 1:** Dropout among patients in methadone treatment during 3 years of following-up in Haiphong, Vietnam (2012–2014)

	0–12 months (*N* = 1055)				13–24 months (*N* = 944)				25–36 months (*N* = 819)				Total dropped out over 3 years	
	Dropped out *N* = 111; *n* (%)		Missed > 5 doses *N* = 32; *n* (%)		Dropped out *N* = 124; *n* (%)		Missed > 5 doses *N* = 32; *n* (%)		Dropped out *N* = 115; *n* (%)		Missed > 5 doses *N* = 68; *n* (%)			
All	111	(10.5)	32	(3.0)	124	(13.1)	32	(3.4)	115	(14.0)	68	(8.3)	350	(33.2)
Reasons for dropout^a^														
Death	21	(18.9)	0	(0.0)	9	(7.3)	0	(0.0)	7	(6.1)	0	(0.0)	37	(10.6)
Arrest	26	(23.4)	0	(0.0)	38	(30.6)	0	(0.0)	20	(17.4)	3	(4.4)	84	(24.0)
Transfer to another clinic	11	(9.9)	0	(0.0)	7	(5.6)	0	(0.0)	7	(6.1)	0	(0.0)	25	(7.1)
Other^b^	12	(10.8)	5	(15.6)	11	(8.9)	7	(21.9)	43	(37.4)	50	(73.5)	66	(18.9)
Not listed	41	(36.9)	27	(84.4)	59	(47.6)	25	(78.1)	38	(33.0)	15	(22.1)	138	(39.4)

^a^Based on clinical records

^b^Family conflicts, side effects, demotivated, and travel

We attempted to locate all 115 persons who had permanently dropped out during their third year of MMT, of whom we were able to locate and interview 28 (24.3%); we also tried to trace all 68 who had missed more than five consecutive doses during the same period, of whom 53 (77.9%) were traced; therefore, we interviewed 81 cases. We also interviewed 161 matched controls still in treatment. Characteristics of cases and controls are shown in Table 2. Almost all (97.9%) clients interviewed were male; slightly less than one half were unemployed. Those who dropped out had slightly lower levels of education, with 71.4% having completed secondary school or less, compared to 50.7% of those who were retained in treatment; they were also more likely to have children (78.7 versus 58.4%). The mean methadone dose of those who permanently dropped out (79.4 mg/day) and those who missed more than five doses (57.1 mg/day) were lower than among those who continued treatment (111.7 mg/day).

**Table 2:** Comparison of those who missed greater than five consecutive doses, who dropped out completely, and who were retained on methadone between 27 and 36 months of treatment in Haiphong (2014)

Variable	Cases				Controls	
	Dropped out *N* = 28; *n* (%)		Missed > 5 doses *N* = 53; *n* (%)		In treatment *N* = 161; *n* (%)	
Demographic characteristics						
Age, years						
24–30	5	(17.9)	6	(11.3)	20	(12.4)
31–40	17	(60.7)	27	(50.9)	85	(52.8)
41–50	6	(21.4)	14	(26.4)	43	(26.7)
51–59	0	(0.0)	6	(11.3)	13	(8.1)
Mean, years (SD)	36.7	(5.5)	39.6	(7.4)	38.4	(7)
Male	27	(96.4)	52	(98.1)	158	(98.1)
Educational level completed						
Primary school	2	(7.1)	3	(5.7)	15	(9.4)
Secondary school	18	(64.3)	20	(37.7)	66	(41.3)
High school	6	(21.4)	28	(52.8)	73	(45.6)
College/university or higher	2	(7.1)	2	(3.8)	6	(3.8)
Marital status						
Single	7	(25)	10	(18.9)	56	(34.8)
Married	17	(60.7)	37	(69.8)	80	(49.7)
Separated/divorced/widowed	4	(14.3)	6	(11.3)	25	(15.5)
Live with family	25	(89.3)	51	(96.2)	153	(95)
Have children	6	(21.4)	12	(22.6)	67	(41.6)
Unemployed	12	(42.9)	23	(43.4)	62	(38.5)
Methadone treatment						
Distance from house to the MMT clinic, mean (SD), km	3.9	(1.9)	5.6	(7.4)	3.9	(3.1)
Most recent methadone maintenance dose, mg/day						
5–59	9	(33.3)	34	(64.2)	43	(26.7)
60–119	11	(40.7)	14	(26.4)	62	(38.5)
120–380	7	(25.9)	5	(9.4)	56	(34.8)
Mean(SD)	79.4	(44)	57.1	(43.5)	111.7	(78.8)
Drug and alcohol use^a^						
Used heroin in the last month of MMT	11	(39.3)	17	(32.1)	9	(5.6)
Used other drugs in the last month of MMT	0	(00)	3	(5.7)	13	(8.1)
Drank alcohol during the last 3 months of MMT	21	(75)	34	(64.2)	101	(62.7)
Medical history		(17.9)				(36.6)
HIV sero-positive	5		8	(15.1)	59	
HbsAg positive	4	(14.3)	5	(9.4)	14	(8.7)
HCV positive	8	(28.6)	25	(47.2)	73	(45.3)
Self-reported mental health problems in the last 3 months^b^	13	(46.4)	38	(71.7)	76	(47.2)
Have current friends who use drugs	13	(46.4)	28	(52.8)	97	(60.2)

^a^Prior to dropping out

^b^Symptoms of anxiety, depression, and suicidal ideation or attempt

Characteristics associated with dropping out either permanently or for more than 5 days are shown in Table 3 and include continuing to use heroin (odds ratio [OR] = 8.92, 95% CI 3.96–20.13, *p* < 0.001) and self-report of a mental health issue (OR = 1.9, 95% CI 1.1–3.29, *p* = 0.021). Those who were not married (OR = 0.49, 95% CI 0.28–0.86, *p* = 0.013) were HIV infected (OR = 0.33, 95% CI 0.33–0.65, *p* = 0.001), and those who received > 60 mg/day of methadone were less likely to drop out. Factors independently associated with dropping out were continuing to use heroin during the last month of MMT (adjusted OR [aOR] = 12.4; 95% CI 4.2–36.8, *p* < 0.01) and receiving a low dose of methadone (<60 mg/day).

**Table 3:** Factors associated with methadone non-compliance (either dropped out or missed < 5 days) during 27–36 months of treatment in Haiphong (2014); *N* = 242

Variable	Crude OR (95% CI)		*p*	Adjusted OR (95% CI)		*p*
Age, years						
24–40	Ref					
41–59	0.89	(0.50–0.56)	0.677			
Gender						
Female	Ref.					
Male	0.75	(0.12–0.58)	0.755			
Educational level completed						
Secondary school and lower	Ref.					
High school and higher	0.91	(0.53–0.55)	0.718			
Marital status						
Married	Ref.			Ref		
Single/widowed/divorced/separated	0.49	(0.28–0.86)	0.013	0.67	(0.24–1.86)	0.440
Have children						
No	Ref.			Ref		
Yes	2.50	(1.36–0.59)	0.003	2.84	(0.88–9.14)	0.081
Distance from house to the MMT clinic, mean (SD), km	1.06	(0.99–0.13)	0.054	1.07	(0.96–1.20)	0.228
Most recent methadone dose (mg/day)						
5–59	Ref.			Ref		
60–119	0.40	(0.22–0.76)	0.005	0.40	(0.17–0.94)	0.036
120–380	0.36	(0.10–0.46)	< 0.001	0.28	(0.09–0.86)	0.026
Number of doses of methadone missed, last 3 months						
No missed doses	Ref.			Ref		
1–3 doses	3.61	(1.66–7.87)	0.001	2.21	(0.86–5.66)	0.098
> 3 doses	24.4	(10.83–4.98)	< 0.001	18.48	(7.25–47.09)	< 0.001
Used heroin during last month of MMT						
No	Ref.			Ref		
Yes	8.92	(3.96–20.13)	< 0.001	12.40	(4.19–36.75)	< 0.001
Have current friends who use drugs						
No	Ref.			Ref		
Yes	0.68	(0.39–1.16)	0.154	0.62	(0.29–1.31)	0.207
HIV status						
Negative	Ref.			Ref		
Positive	0.33	(0.17–0.65)	0.001	1.06	(0.39–2.93)	0.907
Self-reported mental health problems, last 3 months						
No	Ref.			Ref		
Yes	1.90	(1.10–3.29)	0.021	0.99	(0.45–2.17)	0.983

Among the 81 clients who dropped out and were interviewed, the most common reason given for discontinuing treatment was lack of continued dependence on heroin (22.2%) (Table 4). Many also felt that going to the clinic on a daily basis interfered with their work (12.0%), 16.0% had a health problem that prevented regular attendance, and 14.8% stated that they were unable to afford the cost of treatment.

**Table 4:** Reasons for quitting methadone maintenance treatment, based on individual interviews^**^

Reasons	Dropped out *N* = 28 *n* (%)		Missed > 5 doses *N* = 53 *N* (%)		All *N* = 81 *N* (%)	
I am not dependent on heroin anymore	12	(42.9)	7	(13.2)	19	(23.5)
Intentionally want to quit dependence on methadone	0		4	(7.5)	4	(4.9)
Cannot tolerate side effects of methadone	1	(3.6)	1	(1.9)	2	(2.5)
Demotivated, methadone treatment is too long	1	(3.6)	1	(1.9)	2	(2.5)
Time required for MMT conflicts with work	4	(14.3)	12	(22.6)	17	(21.0)
Cannot afford methadone fee	6	(21.4)	6	(11.3)	12	(14.8)
Have a health problem and cannot get to clinic	1	(3.6)	12	(22.6)	13	(16.0)
Gave birth/unavailable for methadone	0		1	(1.9)	1	(1.2)
Family conflict	1	(3.6)	3	(5.7)	4	(4.9)
Moved away	1	(3.6)	0		1	(1.2)
Extended travel away from city	0		3		3	
Arrested	0		3		3	
No reason given	1	(3.6)	0		1	(1.2)

^**^Based on interviews with 81 persons who dropped out between 25 and 36 months

## Discussion

This is one of the few studies to systematically evaluate long-term dropout of clients in methadone treatment in Vietnam. Two thirds of all patients who initiated treatment remained in the program after 3 years. Since the rollout of MMT in Vietnam, only a few other studies have evaluated dropout rates, but they have been over short periods of follow-up [8, 19]. A 2009 study of MMT clinics in Haiphong and Ho Chi Minh City found 10% of clients failed to return during the first 9 months, similar to the 11% dropout we found during the first year [5, 19]; another study of six pilot MMT programs found a 2-year dropout of 22% [5]. Most studies from other countries in the region also report retention during the first year, except for an evaluation conducted in China that followed clients for up to 6 years; the proportion of clients who remained in treatment after 36 months was 66%, similar to our study [17, 20].

Our 1-year dropout rate was considerably lower than that reported from other countries in the region and from some western countries [16, 18, 21, 22]. In Malaysia, 38% of clients dropped out by 12 months [16], and in China, 73% failed to be retained after 1 year [18, 23]. Others studies from the west and Israel found 1-year dropout rates to range from 27 to 40% [22, 24, 25]. In contrast, a study from Germany found 6-year dropout rates to be very low, less than 25%, but this could be attributed to the fact that buprenorphine as well as methadone were available and that most clients received treatment from private physicians’ offices [26]. Retention of clients in methadone programs in Vietnam may be higher than in other countries because of the careful vetting of Vietnamese drug users before they are allowed to access treatment [4, 8]. Those permitted to enter methadone programs must demonstrate stable family or other support systems that a priori may ensure their adherence. Potential clients must also have identity cards and be officially certified as drug users with the local authorities. These policies deter less stable individuals from seeking treatment, including internal migrants, those who are indigent, and those involved in any type of criminal activity. The selection process for MMT in Vietnam has been slightly relaxed since mid-2015; prospective clients are now required to present only a household registration or an identification card but still need to pay some of the cost of methadone treatment. In Haiphong, MMT clinics have recently removed the requirement for identity cards, and this has helped to increase the number of PWID who are receiving drug substitution.

We found that the factors most strongly associated with MMT dropout were continued use of heroin while taking methadone and being administered a methadone dose of less than 60 mg/day. Numerous studies have demonstrated relationships between both continued drug use and inadequate methadone dosing and poor retention. Higher doses of methadone have been shown to be better at preventing PWID from using illicit opiates while on treatment and improving adherence to treatment [17, 20, 27–34]. The US National Institutes of Health recommends 60 mg/day as the lowest effective dose for opiate substitution [35]. Given that one third of patients who dropped out in our study were on less than this amount, Vietnam’s guidelines for methadone dosing should be re-evaluated.

In our study, the primary reasons for non-compliance given by those who had dropped out included conflict with work and the requirement to attend clinic on a daily basis. In many MMT programs in other countries, methadone can be provided to clients for days at a time or even weeks if they are stable. In Vietnam, however, clients must go to the clinic 7 days a week to receive treatment or even twice a day if dosing is divided to reduce drug interactions. These requirements can be burdensome, particularly because clients can only access the MMT clinic in the same district where they have permanent residence. To address these issues, the Vietnam MMT program is considering changing its guidelines to make buprenorphine available which could allow clients to come for refills every several weeks. [36]. In addition, Vietnam may also start using electronic patient cards or fingerprint scanning to help track patients between clinics and thereby allow them to receive methadone at a variety of sites. Other means of improving adherence could involve co-location of MMT and other health services, including HIV treatment clinics [14, 37]. Better integration of health services in Vietnam has been found to improve quality of life, satisfaction with services, and improved retention on ART among those who were HIV infected [37, 38].

Many participants in our study who dropped out also mentioned that the $15 monthly co-payment was excessively burdensome. Overall, Vietnam is experiencing a national shortfall in funding for HIV-related services, including those for PWID [14, 39]. To help off-set deficits, the provinces, which are responsible for running the MMT clinics and paying for staff, can levy a fee on clients; the methadone itself is provided by the national government. Part of the rationale for the fee was based on estimating that $15 was less than 5% of what most PWID spent on illicit opiates per month. In addition, an evaluation during the initial rollout of MMT in Haiphong found that among clients surveyed, $15 per month was considered acceptable [14]. However, these costs need to be included in out-of-pocket health care costs which may also need to be paid by PWID and their families, given the considerable level of co-morbidity among drug users [40]. The greater integration of MMT with other health services, as well as a sliding scale for co-payment, have been suggested as a way to address the economic burden of treatment [40].

Our study had several limitations. We found it difficult to trace clients who had permanently dropped out and were only able to interview one quarter of them, resulting in a loss of representativeness. The majority of clients whom we traced were those who had missed more than five consecutive doses and had re-entered the program; these persons may be fundamentally different from those who have permanently discontinued treatment. Clients were asked structured questions about reasons for dropping out which did not allow us to explore these or other issues that may have been of equal or greater importance.

Nevertheless, we demonstrated that 3-year retention rates were high among the MMT clients in Haiphong and were able to identify several factors that could have an important impact on the long-term efficacy of methadone treatment. Programs should consider sliding fees for co-payment, so that clients should not be expected to subsidize the cost of running the clinics, preventing them from being able to continue treatment. Making methadone available to stable clients for longer periods of time would facilitate job retention and travel. The use of electronic record systems, linking of facilities, and unique identification of clients would allow those in treatment to receive care throughout the province or the country, further enabling retention. These changes, as well as removing the barriers to being eligible to receive methadone in the first place, could go a long way in improving the number of PWID who can receive opiate substitution therapy. This would reduce the substantial burden not only of drug use but also of the HIV epidemic in Vietnam.

## Conclusion

By 3 years, one third of patients under methadone treatment had permanently dropped out. Ensuring that methadone dosing is adequate, reconsidering the co-payment fee, and allowing methadone up to several days so clients can maintain their jobs could improve retention. Introducing buprenorphine in the program would be a good alternative to overcome the weak points above of methadone.

## Abbreviations

aOR: Adjusted odds ratio
MMT: Methadone maintenance therapy
OR: Odds ratio
PWID: Persons who inject drugs
USD: US dollars
VND: Vietnam Dong
